# Real-World Clinical Effectiveness and Safety of Antifibrotics in Progressive Pulmonary Fibrosis Associated with Rheumatoid Arthritis

**DOI:** 10.3390/jcm13237074

**Published:** 2024-11-22

**Authors:** Javier Narváez, Martí Aguilar-Coll, Vanesa Vicens-Zygmunt, Juan José Alegre, Guadalupe Bermudo, María Molina-Molina

**Affiliations:** 1 Department of Rheumatology, Hospital Universitario de Bellvitge, Bellvitge Biomedical Research Institute (IDIBELL), 08907 Barcelona, Spain; maguilarco@bellvitgehospital.cat; 2 Interstitial Lung Disease Unit, Department of Pneumology, Hospital Universitario de Bellvitge, Bellvitge Biomedical Research Institute (IDIBELL), 08907 Barcelona, Spain; vvicens@bellvitgehospital.cat (V.V.-Z.);; 3 Department of Rheumatology, Hospital Universitario Dr. Peset, 46017 Valencia, Spain; txantxo@gmail.com

**Keywords:** rheumatoid arthritis, interstitial lung diseases, antifibrotics, nintedanib, pirfenidone

## Abstract

**Background/Objectives**: Interstitial lung disease (ILD) is one of the most severe complications of rheumatoid arthritis (RA). Real-world data on antifibrotic treatment are needed. Our objective was to evaluate the real-world effectiveness and tolerability of antifibrotic agents in patients with progressive fibrosing RA-ILD. **Methods:** A longitudinal, retrospective, observational study was conducted on a cohort of RA-ILD patients treated with either nintedanib or pirfenidone. The data collected included pulmonary function test (PFT) results, adverse events (AEs), tolerability, and drug retention. **Results:** Twenty-seven patients were included; 25 (92.5%) initiated nintedanib, while two initiated pirfenidone. The median follow-up duration was 25 months (IQR 7–27). The mean decline in %pFVC and %pDLCO from ILD diagnosis to the initiation of antifibrotic therapy were −8.9% and −14.8%, respectively. After 6 months of treatment, most patients achieved stabilization in PFT: a ∆%pFVC of +1.2% (*p* = 0.611 compared with baseline) and a ∆%pDLCO of +3.9% (*p* = 0.400). Eighteen patients completed one year of therapy, with a modest improvement in %pFVC (+4.7%; *p* = 0.023) and stabilization in %pDLCO (−3.8%; *p* = 0.175). This trend persisted among the nine patients who completed 2 years of treatment (%pFVC +7.7%; *p* = 0.037 and %pDLCO −2.2%; *p* = 0.621). During the follow-up period, 15% of patients died, and 4% underwent lung transplantation. Adverse events occurred in 81% of patients, leading to discontinuation in 18.5% of cases. The most frequent adverse events were gastrointestinal events and hepatitis, leading to a permanent dose reduction of 40% for nintedanib and 14% for pirfenidone. A second antifibrotic agent was prescribed for 18.5% of the patients. At the end of the follow-up period, 63% of the total cohort remained on antifibrotic therapy. **Conclusions:** According to our results, antifibrotic initiation was associated with a modest improvement in the trajectory of %pFVC and stabilization in %pDLCO. The discontinuation rate in our cohort (37%) was higher than that reported in clinical trials but similar to that reported in previously published real-world studies.

## 1. Introduction

Interstitial lung disease (ILD) is one of the most common and severe extra-articular manifestations of rheumatoid arthritis (RA) and significantly contributes to both morbidity and mortality [1,2,3,4,5,6,7]. The most prevalent ILD subtypes among RA patients are usual interstitial pneumonia (UIP) and nonspecific interstitial pneumonia (NSIP). The UIP pattern observed in RA-ILD closely resembles idiopathic pulmonary fibrosis (IPF), and both are associated with a high risk of progressive pulmonary fibrosis (PPF) and increased short-term mortality. Additionally, some RA patients with NSIP-type ILD may also develop progressive fibrosing patterns [7].

The clinical trajectory of RA-ILD varies widely. Approximately 55% of RA-ILD patients experience disease progression [8,9], with an estimated 40% meeting the criteria for PPF within 5 years of onset, characterized by a rapid decline in lung function, progression to chronic respiratory failure, and an increased risk of premature mortality [10,11]. In patients with a fibrosing phenotype, the same profibrotic pathways that lead to pulmonary fibrosis in IPF are activated [12,13]. Once these pathways are triggered, they follow an autonomous and self-perpetuating clinical course [12,13]. Inhibiting these activated profibrotic pathways with antifibrotic agents, such as nintedanib or pirfenidone, remains the only strategy to slow or potentially stop fibrosis progression.

Nintedanib has been formally approved by health authorities for the treatment of progressive fibrosing ILD, regardless of its underlying cause, based on the INBUILD trial, which demonstrated a significantly reduced annual rate of forced vital capacity (FVC) decline in these patients [14]. In a focused evaluation of the INBUILD study comparing the rate of decline in % FVC over 52 weeks among patients with autoimmune-related ILD, no significant differences were observed based on the HRCT pattern. The adjusted difference for the UIP-like fibrotic pattern was 124.2 (95% CI: 31.1 to 217.4), compared to 41.7 (95% CI: −112.2 to 195.5) for other fibrotic patterns (*p* = 0.37) [15]. Subanalyses of the 89 patients with RA-ILD in the INBUILD trial revealed similar efficacy to that observed in other causes of PF-ILD [16].

Pirfenidone was evaluated in RA-ILD patients in the TRAIL1 trial, a placebo-controlled study with limited statistical power [17]. Although the primary endpoint (a composite of ≥10% decline in percent predicted FVC (%pFVC) or death within one year) was not met, pirfenidone was associated with a significantly slower rate of FVC decline compared to placebo. Post hoc analyses revealed that the effect of pirfenidone on the decline in FVC was more significant in patients with a UIP pattern [17]. Based on these findings, the 2023 guidelines from the American College of Rheumatology (ACR) and the American College of Chest Physicians (CHEST) for the management of ILD in systemic autoimmune rheumatic diseases conditionally recommend adding pirfenidone as a treatment option for RA-ILD patients with progression despite first-line treatment [18].

Although nintedanib and pirfenidone are effective in slowing the progression of ILD, both drugs are associated with adverse events (AEs) that may restrict their use, often resulting in dose adjustments or discontinuation. While clinical trials provide valuable insights, real-world studies are particularly important because they reflect a broader and more representative patient population typically encountered in clinical practice. Clinical trials frequently exclude patients with advanced disease stages or prevalent comorbidities, while real-world studies not only encompass these individuals but also offer the opportunity for extended follow-up periods.

This study aims to bridge this gap by evaluating the effectiveness and tolerability of antifibrotic therapy in a real-world cohort of patients with RA-ILD PFF.

## 2. Methods

### 2.1. Study Sample

We conducted a review of medical records and hospital pharmacy-prescribing databases to identify all RA patients who initiated nintedanib or pirfenidone as part of routine clinical care at two tertiary referral hospitals (Bellvitge University Hospital and Dr. Peset University Hospital, Spain). Antifibrotic therapies were prescribed in all cases due to progressive fibrosing RA-associated interstitial lung disease (RA-ILD), and no patients were excluded based on poor outcomes or early death.

In line with previous studies from our group [19,20,21], progressive ILD was defined by one or more of the following criteria during follow-up: a relative decline of ≥10% in the %pFVC or ≥15% in the predicted diffusing capacity for carbon monoxide, corrected for hemoglobin (%pDLCO) during follow-up, or a relative decline of 5–10% in %pFVC accompanied by a reduction of less than 15% in %pDLCO, together with worsening respiratory symptoms and increased fibrosis as assessed by thoracic high-resolution computed tomography (HRCT) [14,22]. These changes occurred within 2 years of ILD diagnosis [14].

RA was diagnosed according to the ACR 1987 classification criteria [23] or the ACR/EULAR 2010 criteria [24], depending on the year of diagnosis. ILD was identified using HRCT of the chest, with experienced thoracic radiologists classifying cases into three main radiologic patterns based on the American Thoracic Society (ATS)/European Respiratory Society (ERS) International Multidisciplinary Consensus Classification of Idiopathic Interstitial Pneumonias [25]: (1) UIP; (2) NSIP, and (3) other patterns. The final diagnosis of progressive fibrosing RA-ILD was confirmed in all patients through a multidisciplinary approach.

Patients were managed according to a standardized protocol within a specialized multidisciplinary unit, overseen jointly by a pulmonologist and a rheumatologist. Pre-existing treatments, including glucocorticoids (GCs), conventional synthetic disease-modifying antirheumatic drugs (csDMARDs), immunosuppressants (ISs), and biologic DMARDs (bDMARDs) initially remained unchanged in all patients. However, adjustments to GC dosages were made as needed after the initiation of antifibrotic therapy, at the discretion of the treating physician.

Pulmonary function tests (PFTs) were conducted every 6 months following the initiation of treatment. All tests were performed in a standardized manner, following the 2002 recommendations of the Spanish Society of Pneumology and Thoracic Surgery [26]. Both %pFVC and %pDLCO were measured simultaneously for each patient.

### 2.2. Clinical Assessments and Outcome Variables

The effectiveness of antifibrotic therapies was assessed by evaluating changes in %pFVC and %pDLCO before and after treatment initiation. The progression of PFTs was categorized according to the definitions established by the ATS as worsening (a decrease in %pFVC > 10% or %pDLCO > 15%), stabilization (changes in %pFVC < 10% or %pDLCO < 15%), or improvement (an increase in %pFVC > 10% or %pDLCO > 15%) [27,28].

Data regarding antifibrotic therapies included the specific drug used, dosage, duration of follow-up from the first dose, treatment status at the endpoint (continuation or discontinuation), reasons for discontinuation (if applicable), tolerability, and side effect profile. Additional information on lung transplants and deaths (including causes of death) was also collected. The endpoint for follow-up was defined as the date of the last clinic visit, death, or lung transplant. A retrospective analysis of prospectively collected data was performed.

### 2.3. Statistical Analysis

The results are expressed as the mean ± standard deviation (SD) or as the median (interquartile range [IQR], 25th–75th) as appropriate for continuous data, whereas categorical variables are presented as the number of cases and percentages.

The Kolmogorov–Smirnov test was employed to determine whether numerical variables followed a normal distribution. Depending on the distribution, numerical variables were compared using either the Student’s *t*-test or the Mann–Whitney U test. For categorical variables, the chi-squared test or Fisher’s exact test was applied, as appropriate.

Pulmonary function trends were quantified as a percentage change (delta) from the time of diagnosis of RA-ILD to the time of initiation of antifibrotic treatment (T0) and in relation to T0 for all subsequent evaluations after starting therapy. The paired sample t- test was used to compare pre- and post- antifibrotic treatment means of the main outcome efficacy measures evaluated. Statistical significance was defined as *p* < 0.05.

## 3. Results

### 3.1. Patient Characteristics

To date, antifibrotic therapies have been administered to 27 patients with ongoing progressive fibrosing RA-ILD despite prior immunosuppressive treatment. The key baseline characteristics of this cohort are summarized in Table 1. The mean age at the start of antifibrotic therapy was 67 ± 10 years. At that time, the median duration of RA was 70.5 months (interquartile range [IQR], 25th–75th percentile: 27.5–114 months), while the median time since ILD diagnosis was 29 months (IQR: 20–50 months).

Anticitrullinated protein antibodies (ACPA) were positive in 81.5% of patients, and rheumatoid factor (RF) in 85%. Radiologically, 21 patients (78%) were classified as having UIP, five (18%) as fibrotic NSIP, and one (4%) as combined pulmonary fibrosis and emphysema (CPFE). A smoking history was reported in 18 patients (67%).

With respect to antifibrotic treatment, 25 (92.5%) patients initiated therapy with nintedanib, while two started therapy with pirfenidone due to concurrent anticoagulant use. During follow-up, nintedanib was switched to pirfenidone in five patients because of adverse effects; thus, seven (26%) patients received pirfenidone at some point during the follow-up period.

At the onset of antifibrotic therapy, 10 (37%) patients exhibited moderate to high RA activity according to the DAS28 ESR score (>3.2). The mean %pFVC was 86.6 ± 15 (IQR 25th–75th percentile, 73–97.1), and the mean %pDLCO was 54.3 ± 14.8 (IQR 44–66). Fourteen (52%) patients presented a decline of ≥10% in %pFVC.

### 3.2. Treatment Characteristics

Prior to the initiation of antifibrotic therapy (see Table 1), 26 (96%) patients had been treated with GCs, 22 (82%) had received at least one csDMARD or IS, and 20 (74%) had been treated with one or more bDMARDs.

Previous csDMARDs and IS included leflunomide (LEF) in 18 (67%) patients, methotrexate (MTX) in seven (26%), sulfasalazine in two (8%), and mycophenolate mofetil (MMF) in two (8%). The previous bDMARDs administered were rituximab (RTX) in 10 (37%), abatacept (ABA) in 11 (41%), anti-tumor necrosis factor (TNF) alpha agents in four (15%), and tocilizumab in one (4%).

All patients received antifibrotic therapy combined with either a bDMARD, a csDMARD/IS, or both: 17 (63%) received a bDMARD plus a csDMARD/IS, 5 (19%) received only a csDMARD/IS, and five (19%) were on bDMARD monotherapy. Additionally, 25 (93%) patients also received GC at a mean dose of 8.3 ± 5.4 mg/day (IQR 5–10).

The biologic agents initially administered at the start of antifibrotic therapy were ABA in 11 (41%), RTX in 8 (30%), JAK inhibitors (JAKis) in one (4%), and adalimumab in one (4%). During follow-up, ABA was replaced by a JAKi in one patient, and RTX was exchanged with ABA or vice versa in three patients (two initially treated with ABA were switched to RTX, and one initially treated with RTX to ABA). The immunosuppressive treatments used were LEF in 16 patients (59%), MMF in four (15%), and MTX in one (4%).

Two patients (7.4%) had type 3 pulmonary hypertension confirmed by right heart catheterization and seven patients (26%) required oxygen therapy at the start of antifibrotic therapy. The median follow-up period after initiating antifibrotic agents was 25 months (IQR 7–27), with a total follow-up of 48.7 patient years.

### 3.3. Efficacy Endpoints

**Figure 1:**
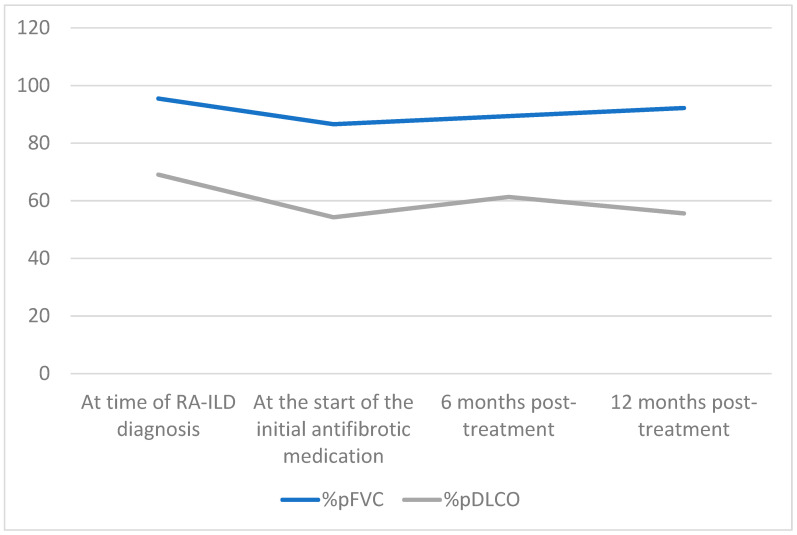
Evolution of the predicted forced vital capacity (%pFVC) and the predicted diffusing capacity for carbon monoxide corrected for hemoglobin (%pDLCO) before initiation of antifibrotic therapy and after 1 year of treatment.

Changes in the primary efficacy outcome measures assessed at 6 months and 1 year following the initiation of antifibrotic therapy are presented in Table 2 and Table 3 and illustrated in Figure 1. Across the entire study population, prior to starting therapy, the mean decline in %pFVC and %pDLCO from the time of ILD diagnosis to the initiation of antifibrotic treatment (T0) was −8.9% (95% CI: 7.81 to 16.02; *p* = 0.0001) and −14.8% (95% CI: 9.53 to 20.16; *p* = 0.0001), respectively.

In three patients, antifibrotic therapy was discontinued due to adverse effects before completing 6 months of treatment. In the remaining 24 patients (twenty-one UIP and three non-UIP patterns), a slower decline in PFT parameters was observed after 6 months of therapy (delta: percentage change from baseline): ∆%pFVC + 1.2% (95% CI: −5.67 to 3.41; *p* = 0.611 compared with T0) and ∆%pDLCO + 3.9% (95% CI: −13.51 to 5.63; *p* = 0.400).

At 1 year of treatment, data were unavailable for nine patients: three had died due to ILD progression, four had discontinued treatment due to adverse effects, and two had not yet completed 12 months of therapy. Among the 18 patients who completed one year of therapy (15 UIP and three NSIP patterns), a modest improvement in %pFVC was observed (∆ + 4.7%, 95% CI: −8.66 to −0.74; *p* = 0.023), along with a slowing in %pDLCO decline (∆ − 3.8%, 95% CI: −1.82 to −9.25; *p* = 0.175).

Nine of the 24 patients completed 2 years of treatment (seven UIP and two NSIP), maintaining a response in PFTs: ∆%pFVC: +7.7% (95% CI: −16.07 to −0.66; *p* = 0.037) and ∆%pDLCO: −2.2% (95% CI: −7.67 to −12.07; *p* = 0.621).

**Figure 2:**
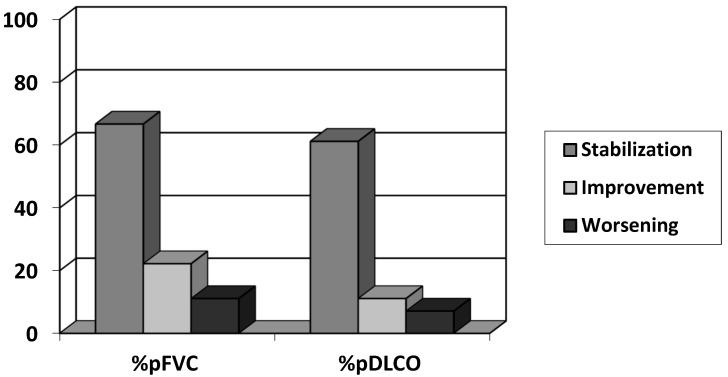
Lung function test results (as defined by the ATS) after 12 months of antifibrotic therapy.

A comparison of pre- and post-treatment pulmonary variables revealed that PFT deterioration either slowed or stabilized in approximately three quarters of the patients (Table 3 and Figure 2). Additionally, prednisone doses were reduced in 10 out of 25 patients (40%) following the initiation of antifibrotic therapy, with a mean reduction of −5.8 mg/day (SD: 3.5 mg; 95% CI: 7.73 to 15.62; *p* < 0.046). Efficacy comparisons between radiological patterns could not be performed due to the low number of non-UIP patterns.

### 3.4. Antifibrotic Tolerability and Retention

Seventeen patients (63%) continued treatment after a median follow-up period of 25 months (IQR 7–27) following the initiation of antifibrotic therapy. Among the 10 patients who discontinued treatment, the reasons were death due to ILD progression and infectious complications in four patients (15%), lung transplantation in one patient (4%), and adverse events leading to treatment discontinuation in five patients (18.5%). The retention rate of patients initially treated with nintedanib was 52% (13/25).

A total of 81.5% (22/27) of patients experienced adverse events (AEs) attributed to antifibrotic treatment. Their frequency and types are detailed in Table 4. As expected, the most common events were gastrointestinal events and hepatitis.

In 48.1% (13/27) of patients, antifibrotic therapy was temporarily suspended (mean number of suspensions: 1.38 ± 0.5; range: 1–2), and in 63% (17/27) of patients, the recommended dosage was temporarily reduced (mean number of dose reductions: 1.53 ± 0.5; range: 1–3).

The most frequent AEs leading to treatment discontinuation or permanent dose reduction were diarrhea and hepatitis.

As previously reported, a second antifibrotic agent was prescribed for five (18.5%) patients; all were initially treated with nintedanib and were switched to pirfenidone due to adverse events. Among these five patients, three (60%) remained on pirfenidone after a median follow-up of 13 months.

## 4. Discussion

To date, the effectiveness and tolerability of antifibrotics in RA-ILD have been evaluated in two randomized controlled trials (INBUILD and TRAIL1) [16,17], three observational studies [29,30,31], and several case reports and case series [32,33,34,35,36]. Our study is among the few real-world analyses assessing antifibrotic effectiveness and drug retention in RA-ILD patients. Real-world data enable the inclusion of a more diverse patient population, making the findings more reflective of daily clinical practice. Additionally, real-world studies offer valuable insights into long-term safety, especially concerning rare adverse events.

In both clinical trials (INBUILD with nintedanib and TRAIL1 with pirfenidone), antifibrotic treatment demonstrated only a significant reduction in the rate of FVC decline, without stopping or improving its value [16,17]. In contrast, real-world studies appear to demonstrate either stabilization or a modestly improved trajectory in %pFVC [29,30,31].

In our cohort, the initiation of antifibrotics was associated with an initial stabilization in %pFVC at 6 months, followed by a gradual, modest improvement (+4.7%; *p* = 0.023) after the first year of therapy, whereas %pDLCO showed a mild, non-significant decline (−3.8%; *p* = 0.175), indicating stabilization. The discrepancy between our results and those of the clinical trials may be explained by selection bias, as all our patients were worsening prior to starting antifibrotic treatment, which may bias outcomes toward improvement or stabilization after this time point.

Duarte et al. [30] also reported that antifibrotic therapy interrupted the decline in FVC, shifting from a pre-treatment decrease of 300 ± 500 mL per year to an improvement of 200 ± 400 mL in the year following treatment initiation (*p* = 0.336), whereas %pDLCO continued to decline slightly (3% pre-treatment vs. 2.9% post-treatment, *p* = 0.75). In Juge et al.‘s study, both nintedanib and pirfenidone were associated with a reduced decline in the FVC and DLCO trajectories over 18 months [29]. This trajectory change was significant for %pFVC (0.3% per year post-initiation compared with 6.2% per year pre-initiation, *p* = 0.03) but not for absolute FVC or DLCO. In Behera’s study, after 6 months of antifibrotic treatment, the lung function values stabilized, with the %FVC from 62.5 ± 20.04 at baseline to 63.2 ± 18.2 (*p* = 0.3) and the %DLCO increasing from 70.1 ± 15.2 to 72.1 ± 12.4 (*p* = 0.15) [31].

Recently, Ushio et al. have further demonstrated that nintedanib combined with ISs significantly improves %FVC in patients with connective tissue disease-associated progressive fibrosing ILD, including cases with RA-ILD. Before treatment, the mean monthly decline in FVC was −0.70%/month. After therapy, this shifted to a mean increase of +0.54%/month (*p* < 0.001), with greater improvements observed in the nintedanib+IS group (+1.71%/month) compared to the nintedanib monotherapy group (+0.34%/month). Additionally, serum KL-6 levels, a biomarker of fibrosis, decreased significantly. The authors concluded that nintedanib improves %FVC in connective tissue disease-associated PF-ILD, with the combination of nintedanib plus ISs proving more effective than nintedanib monotherapy in stabilizing pulmonary function [32].

Despite the apparent beneficial effect of antifibrotic therapy, 15% of our patients died, and 4% underwent lung transplantation during the follow-up period. In other observational studies, mortality rates ranged from 22% to 35% [29,30], whereas Juge’s study reported a lung transplant rate of 5% [29], which closely aligns with our findings. Given the persistently high rates of lung transplant and mortality, it may be worth reconsidering whether antifibrotics are being introduced too late in clinical practice and assessing the potential benefit of initiating them earlier in patients with fibrotic ILD patterns.

Real-world data confirm the safety of antifibrotic treatments combined with glucocorticoids, csDMARDs/ISs (LEF, MTX, and MMF), with or without biologic agents (particularly ABA and RTX) or JAK inhibitors, without any observed increase in serious infection risk [29,30,31,32,33,34,35,36]. AEs occurred in 81% of our patients, which is higher than the 25% to 55.4% reported in other observational studies [29,30,31], but lower than the 100% reported in the INBUILD and TRAIL 1 trials [16,17]. As in other studies, the most common AEs were gastrointestinal events and hepatitis.

In our series, adverse events resulted in a permanent dose reduction for 40% of patients on nintedanib and 14% on pirfenidone and led to the discontinuation of antifibrotic therapy in 18.5% of patients (compared with 21.4% of RA-ILD patients treated with nintedanib in the INBUILD trial [16] and 24% of patients treated with pirfenidone in the TRAIL1 trial [17]).

However, owing to this and other factors (such as death from ILD progression and infectious complications, or the need for lung transplantation), the retention rate at the end of the follow-up period was only 63% (52% for nintedanib). The discontinuation rate in our cohort was 37% overall and 48% among those initially treated with nintedanib, which was higher than that reported in clinical trials (INBUILD: 23.8%, TRAIL1: 24%), but similar to that reported in previously published real-world studies (30% to 46%) [29,30].

Finally, on the basis of our experience with both RA-ILD and systemic sclerosis-associated ILD, switching to pirfenidone may be a successful alternative for patients intolerant to nintedanib. In our series, efficacy comparisons between radiological patterns could not be performed due to the low number of non-UIP patterns. However, in the study by Juge et al. [29], both nintedanib and pirfenidone demonstrated similar changes in FVCpp trajectory, with no statistically significant differences.

When interpreting our study’s results, it is crucial to acknowledge several limitations inherent to its retrospective observational design. These include the small sample size, selection bias (as all patients had experienced ILD worsening prior to initiating antifibrotic therapy), the absence of post-treatment HRCT, the concomitant use of oral GCs, csDMARDs/ISs, and/or biologics in all patients, and the lack of a control group. While the effects on lung progression cannot be definitively attributed solely to antifibrotics, the study specifically included patients with progressive RA-ILD who had not responded to conventional therapies. Furthermore, the baseline regimens of csDMARDs, ISs, and/or biologic agents were maintained without de-escalation throughout the follow-up period after antifibrotic initiation, allowing patients to serve as their own controls. Pre- and post-treatment lung function trends provided compelling evidence of treatment effects attributable to antifibrotic rescue therapy, a conclusion further supported by the use of objective outcome measures, which minimized examiner bias. Nonetheless, we cannot rule out other residual confounders affecting associations between antifibrotics use and outcomes, since no adjusted analysis could be performed due to the small sample size.

However, our data reflect outcomes from real-world clinical practice in managing refractory cases of this severe complication, with the added strength that our study was conducted independently of corporate sponsorship. Additionally, patients were followed in a structured, protocolized manner with standardized data collection, and the duration of antifibrotic exposure was relatively prolonged.

In conclusion, our real-world data indicate that antifibrotic therapy stabilizes lung function in most patients with progressive fibrosing RA-ILD. Adverse events are frequent, particularly gastrointestinal events, which impact treatment survival and lead to a nonnegligible rate of permanent dose reduction and treatment discontinuation. Further studies with larger sample sizes are necessary to strengthen these findings, particularly regarding effectiveness analysis, and to better establish the optimal therapeutic window for their use.

## Figures and Tables

**Table 1:** Characteristics of patients with RA-ILD at the initiation of initial antifibrotic medication.

Demographics and RA Characteristics	N = 27
Age at age at initiation of antifibrotic treatment, years (mean ± SD)	67 ± 10
Sex, woman/man	12 (44.5%)/15 (55.5%)
Body mass index (BMI), missing data = 4 (mean ± SD)	28.3 ± 5.3
Smoker or ex-smoker	18 (67%) Current: 1 (4%)
Positive rheumatoid factor	23 (85%)
Positive ACPA	22 (81.5%)
Median duration of RA, months (IQR 25th–75th)	70.5 (27.5–114)
DAS28-ESR at initiation of antifibrotic treatment	
Remission or low activity	17 (63%)
Moderate or high activity	10 (37%)
**ILD characteristics**	
Median duration of ILD, months (IQR 25th–75th)	29 (20–50)
HRCT pattern of ILD	
*Usual interstitial pneumonia (definite or probable)*	21 (78%)
*Fibrotic non-specific interstitial pneumonia*	5 (18%)
*Combined pulmonary fibrosis and emphysema*	1 (4%)
%pFVC at initiation of initial antifibrotic medication	86.6 ± 15
%pDLCO at initiation of initial antifibrotic medication	54.3 ± 14.8
**Prior treatments**	
Glucocorticoids	26 (96%)
Mean dose (± SD), mg/day (IQR 25th–75th)	8.3 ± 5.4 (5–10)
csDMARDs or immunosuppressants *	22 (81.5%)
Number of previous csDMARDs or immunosuppressants	1.4 (minimum 1, maximum 2)
*Methotrexate*	7 (26%)
*Leflunomide*	18 (67%)
*Sulfasalazine*	2 (8%)
*Mycophenolate mofetil*	2 (8%)
bDMARD *	20 (74%)
Number of previous bDMARD	1.5 (minimum 1, maximum 6)
*Rituximab*	10 (37%)
Number of cycles	4.4 (minimum 1, maximum 10)
*Abatacept*	11 (41%)
*TNFi*	4 (15%)
*Tocilizumab*	1 (4%)
**Antifibrotic medication**	
Nintedanib	25 (92%)
Pirfenidone	
As initial antifibrotic medication/After nintedanib	2 (8%)/5 (18.5%)
**Concomitant medication**	
Glucocorticoids	25 (93%)
Dose of prednisone at initiation of initial antifibrotic medication, (mean ± SD)	7.9 ± 5
csDMARDs or immunosuppressants	21 (78%)
*Leflunomide*	16 (59%)
*Mycophenolate mofetil*	4 (15%)
Methotrexate	1 (4%)
bDMARD or JAKi *	22 (81.5%)
*Abatacept*	13 (48%)
*Rituximab*	10 (37%)
*TNFi*	1 (4%)
*JAKi*	2 (8%)
Need for oxygen therapy at initiation of initial antifibrotic medication	7 (26%)

* Some patients received more than one. Abbreviations ACPA: anticitrullinated protein autoantibodies; bDMARD: biologic disease-modifying antirheumatic drugs; csDMARDs: conventional synthetic disease-modifying antirheumatic drugs; DAS28-ESR: Disease Activity Score in 28 joints using Erythrocyte Sedimentation Rate; HRCT: high-resolution computed tomography; ILD: interstitial lung disease; IQR: interquartile range; JAKi: Janus kinase inhibitors; %pDLCO: percent predicted diffusing capacity for carbon monoxide corrected for hemoglobin; %pFVC: percent predicted forced vital capacity; RA: rheumatoid arthritis; SD: standard deviation; TNFi: tumor necrosis factor inhibitor.

**Table 2:** Changes before and after 6 months and after 1 and 2 years of treatment with antifibrotic medication.

Before Antifibrotics				
	At Time of RA-ILD Diagnosis Mean ± SD (IQR, 25th–75th)	At Time of Initiation of Initial Antifibrotic Medication Mean ± SD (IQR, 25th–75th)	Delta (Mean)	*p* Value (95% CI)
Total sample (N = 27)				
%FVC predicted	95.5 ± 14.6 (88.1–107.5)	86.6 ± 15 (73–97.1)	−8.9%	0.0001 (7.81 to 16.02)
%DLCO predicted	69.1 ± 18.8 (55.4–79.5)	54.3 ± 14.8 (44–66)	−14.8%	0.0001 (9.53 to 20.16)
**After 6 months of treatment**				
	**At time of** **initiation of initial antifibrotic medication** **mean ± SD** **(IQR, 25th** **–75th)**	**6 months post-treatment** **mean ± SD** **(IQR, 25th** **–75th)**	**Delta** **(mean)**	***p* value** **(95% CI)**
**Total sample (N = 24)**				
UIP: 21/Non-UIP: 3				
%FVC predicted	88.2 ± 19 (73.7–102.7)	89.4 ± 22.7 (74.4–105.5)	+1.2%	0.611 (−5.67 to 3.41)
%DLCO predicted	57.4 ± 16.6 (46.2–68.1)	61.3 ± 24.9 (40.5–79.6)	+3.9%	0.400 (−13.51 to 5.63)
**After 1 year of treatment**				
	**At time of** **initiation of initial antifibrotic medication** **mean ± SD** **(IQR, 25th** **–75th)**	**12 months post-treatment** **mean ± SD** **(IQR, 25th** **–75th)**	**Delta** **(mean)**	***p* value** **(95% CI)**
**Total sample (N = 18)**				
UIP: 15/NSIP: 3				
%FVC predicted	87.5 ± 20.7 (71.8–108.6)	92.2 ± 24.8 (76–115.8)	+4.7%	0.023 (−8.66 to 0.74)
%DLCO predicted	58.2 ± 17.9 (45.7–69.9)	54.4 ± 16.7 (42.7–67)	−3.8%	0.175 (−1.82 to −9.25)
**After 2 years of treatment**				
	**At time of** **initiation of initial antifibrotic medication** **mean ± SD (IQR, 25th–75th)**	**24 months post-treatment** **mean ± SD (IQR, 25th–75th)**	**Delta** **(mean)**	***p* value** **(95% CI)**
**Total sample (N = 9)**				
UIP: 7/NSIP: 2				
%FVC predicted	89.1 ± 19.7 (71.6–111.4)	97.4 ± 19.7 (84.3–120.1)	+7.7%	0.037 (−16.07 to 0.66)
%DLCO predicted	60.8 ± 20.3 (43.8–81.9)	58.6 ± 15.1 (45.5–73.1)	−2.2%	0.621 (−7.67 to −12.07)

%pFVC = predicted forced vital capacity; %pDLCO = predicted diffusing capacity for carbon monoxide, corrected for hemoglobin; UIP: usual interstitial pneumonia and NSIP: fibrotic non-specific interstitial pneumonia.

**Table 3:** Lung function test results according to the definitions of ATS after antifibrotic therapy.

After 6 Months of Treatment N = 24			
	Improvement	Stabilization	Worsening
%FVC predicted	12.5% (3)	75% (18)	12.5% (3)
%DLCO predicted	21% (5)	58% (14)	21% (5)
After 12 months of treatment N = 18			
%FVC predicted	22.2% (4)	66.7% (12)	11.1% (2)
%DLCO predicted	11.1% (2)	61.2% (11)	27.7% (5)
After 24 months of treatment N = 9			
%FVC predicted	44.5% (4)	55.5% (5)	0
%DLCO predicted	22.2% (2)	44.5% (4)	33.3% (3)

**Table 4:** Adverse events associated with antifibrotic therapy.

	N = 27
Any adverse event	22 (81.5%)
**Most frequent adverse events**	
Diarrhea	17 (63%)
Nausea/Vomiting	9 (33%)
ALT o AST increased	7 (26%)
Decreased appetite/weight loss	10 (37%)
Asthenia	2 (7.4%)
Abdominal pain	3 (11%)
**Adverse event leading to permanent dose reduction**	
Nintedanib (N = 25)/Pirfenidone (N = 7)	10 (40%)/1 (14%)
**Adverse event leading to treatment discontinuation**	5 (18.5%)

## Data Availability

The authors confirm that all data underlying the findings are fully available without restriction. All relevant data are included in the paper.
